# *DRD4*-exonIII-VNTR Moderates the Effect of Childhood Adversities on Emotional Resilience in Young-Adults

**DOI:** 10.1371/journal.pone.0020177

**Published:** 2011-05-27

**Authors:** Debjani Das, Nicolas Cherbuin, Xiaoyun Tan, Kaarin J. Anstey, Simon Easteal

**Affiliations:** 1 John Curtin School of Medical Research, The Australian National University, Canberra, Australia; 2 Center for Mental Health Research, The Australian National University, Canberra, Australia; University of Michigan, United States of America

## Abstract

Most individuals successfully maintain psychological well-being even when exposed to trauma or adversity. Emotional resilience or the ability to thrive in the face of adversity is determined by complex interactions between genetic makeup, previous exposure to stress, personality, coping style, availability of social support, etc. Recent studies have demonstrated that childhood trauma diminishes resilience in adults and affects mental health. The *Dopamine receptor D4* (*DRD4*) exon III variable number tandem repeat (VNTR) polymorphism was reported to moderate the impact of adverse childhood environment on behaviour, mood and other health-related outcomes. In this study we investigated whether *DRD4*-exIII-VNTR genotype moderates the effect of childhood adversities (CA) on resilience. In a representative population sample (n = 1148) aged 30–34 years, we observed an interactive effect of *DRD4* genotype and CA (β = 0.132; *p* = 0.003) on resilience despite no main effect of the genotype when effects of age, gender and education were controlled for. The 7-repeat allele appears to protect against the adverse effect of CA since the decline in resilience associated with increased adversity was evident only in individuals without the 7-repeat allele. Resilience was also significantly associated with approach-/avoidance-related personality measures (behavioural inhibition/activation system; BIS/BAS) measures and an interactive effect of *DRD4*-exIII-VNTR genotype and CA on BAS was observed. Hence it is possible that approach-related personality traits could be mediating the effect of the *DRD4* gene and childhood environment interaction on resilience such that when stressors are present, the 7-repeat allele influences the development of personality in a way that provides protection against adverse outcomes.

## Introduction

Exposure to stress or trauma, although a common life experience, has different individual outcomes ranging from severe post-traumatic psychopathology to successful adaptation with minimal negative impact. Emotional Resilience is a multidimensional characteristic that moderates the influence of stressful life-events on mental health outcomes [1]. Resilience varies with context, age, gender, ethnic background, and even within an individual depending on life circumstances [2]. The mechanisms underlying the development of resilience are far from being completely understood. But it is clear that resilience is determined by complex interactions of a number of factors including genetic constitution, history of stress exposure, individual attributes such as personality, coping style, availability of social support, etc. [3]. Recent research also suggests that resilience levels in individuals could be enhanced through certain forms of cognitive behavioral therapy [3]. Better understanding of resilience could improve recovery from stressful experiences and identify at-risk groups for preventive interventions that promote positive adaption to stress.

Campbell-Sills [4] investigated the effect of demographics and history of childhood adversity (CA) on perceived resilience in the general population using the self-report scale developed by Connor and Davidson (Connor-Davidson Resilience Scale or CD-RISC, [2]). They found that childhood maltreatment alone explained 2% of the variance in resilience in their study sample. This is not surprising since the correlation between childhood trauma and psychiatric disorders is well-established [5]. CA was associated with 44.6% of all childhood-onset disorders and 25.9% to 32.0% of late-onset disorders in a large population-based survey [6]. Furthermore, genetic factors are important moderators of environmental stress during development, and there is strong evidence that the dopamine receptor D4 gene (*DRD4*) is one of the genes that moderates the effect of childhood stress on behavioural traits [7], [8], [9], [10].

The human *DRD4* gene carries a variable number tandem repeat (VNTR) polymorphism in the third exon (exIII). Allelic variants with 1–11 imperfect copies of the tandem repeat have been reported [11], [12]. In European populations the ancestral 4-repeat (4 r) allele is most common. Haplotype variation around the less common, derived 7-repeat (7 r) allele indicates that it has reached its current frequency of ∼20% through the action of natural selection [11], [13]. DRD4 molecules carrying 7 copies of the tandem repeat are less efficient at inhibiting the enzyme adenylate cyclase compared to those carrying 4 copies [14], [15]. The presence of the VNTR was also shown to affect mRNA expression *in vitro* (Schoots 2003). However, in a recent study mRNA levels measured in postmortem brain samples did not differ significantly between carriers vs. non-carriers of the 7 r allele [16]. Since the sample size of the study was small the authors report that the lack of statistically significant functional evidence could have been a result of type 2 error. Therefore, differences in gene expression and/or receptor function remain a plausible underlying cause of the numerous gene-behaviour associations reported for the *DRD4*-exIII-VNTR.

Children with the 7 r allele (7r+) are reported to have significantly more externalizing problems, sensation-seeking behaviour and attachment disorganization compared to children without the 7 r allele (7 r−) when exposed to low parenting quality, maternal insensitivity or maternal unresolved loss or trauma [7], [8], [9], [10]. However, they also have fewer problems when quality of parenting is high [10], leading to the suggestion that *DRD4* is a ‘plasticity gene’ that makes individuals more susceptible to environmental influences, both positive and negative [8].

In this study we have extended Campbell-Sills et al's [4] investigation on the effect of CA and CD-RISC scores by examining whether *DRD4*-exIII-VNTR polymorphism moderates the effect of CA on adult emotional resilience. Given the importance of constitutional variables such as temperament and personality in determining individual resilience levels [1], [3], [17], we also explored the effect of personality traits in this context. To best of our knowledge, the effect of *DRD4* genotype on adult emotional resilience has not been investigated previously in a large, randomly selected, community-based sample.

## Methods

### Ethics statement

The study was approved by the ethics committee of The Australian National University. All participants gave written informed consent to be included in the PATH project.

### Participants

The study sample was drawn from the PATH Through Life Project; a longitudinal study of mental health and ageing [18], [19] in three age groups (20–24, 40–44, 60–64 years at baseline) of randomly selected individuals to be followed-up every four years for 20 years. Participants were residents of the city of Canberra and the adjacent town of Queanbeyan, Australia and were recruited randomly from the electoral roll, which provides a good representative population sample because enrolment to vote is a legal requirement for all adult Australian citizens. Participants were surveyed to access information on health, medication, personality, socio-demographics, cognition, and many other variables. Buccal epithelial cell samples for genetic analysis were collected during the first survey. The present study used data from 20+ cohort at the third wave of data collection (since the CD-RISC scale was introduced in the survey only in this wave), which included 1978 individuals aged 30–34 years. Participants had provided information on experience of childhood adversities at wave 1. Individuals who reported to be of European descent and with specific *DRD4*-exIII-VNTR genotypes (see below) were included in this study. After excluding those with missing data for all variables of interest, a sample size of n = 1148 was available for analyses.

### Genotyping

Genotyping of the *DRD4-*exIII-VNTR for the study sample and analysis of consistency with Hardy-Weinberg Equilibrium (HWE) expectations have been reported in a previous study [12]. Briefly, buccal epithelial cells were used as the source of genomic DNA and the extraction was performed using QIAamp blood kits (QIAGEN, Hilden, Germany). Genotyping was performed following the method described by Li et al. [20] using Forward primer: 5′ GCTGCTGCTCTACTGGGC3′ and Reverse primer: 5′GTGCACCACGAAGAAGGG3′ for the polymerase chain reaction. Ten percent of the sample was genotyped twice for quality control and alleles with >7 repeats were confirmed by sequencing. Genotype frequencies were tested for deviation from HWE using an exact test with likelihood-ratio as the test statistic, as appropriate for a sample containing multiple rare alleles [21]. The ExactoHW software was used for the analysis (http://www.genetics.org/cgi/content/full/genetics.109.108977/DC1).

### Measures

Resilience was measured using Connor-Davidson's Resilience scale (CD-RISC) [2], [22]. CD-RISC has 25 items, each with a 5-point range of responses. The total score ranges from 0 to 100, with higher scores indicating greater resilience [2]. Assessments of CD-RISC in culturally diverse, clinical and general population samples have demonstrated that it is a valid and a reliable measure of resilience (Cronbach's α = 0.89). [2], [23], [24], [25], [26]. Burns et al. [22] have recently reported the psychometric properties of CD-RISC in the present study sample.

Experience of CA up to the age of 16 years was assessed using a 17-item questionnaire as described in earlier studies [27], [28]. The unweighted sum of the 17 items was used to generate the continuous scale for CA [28]. We used only the total number of adversities reported in our analyses without further classifying adversities into specific types.

Approach and avoidance tendencies postulated by Gray [32], [33] to be controlled by the behavioural inhibition and activation system were assessed with a self-report scale developed by Carver and White [29]. Behavioural inhibition system (BIS)/behavioural activation system (BAS) sensitivities were measured with three subscales representing elements of BAS (BAS-drive, BAS-reward response, BAS-fun seeking) and one scale for BIS, which have been validated in culturally diverse samples [18], [29], [30].

In our sample 10 alleles and 25 different genotypes for the *DRD4-*exIII-VNTR polymorphism were identified. The distribution of genotypes did not differ significantly from the HWE expectation [12]. In this study we compared only the most common 4 r and 7 r alleles, since there is evidence of functional differences between these alleles [14], [15]. The functional status of other, rare alleles has not been experimentally determined. We recently compared the different schemes commonly used to group *DRD4-*exIII-VNTR alleles and showed that phenotypic associations identified for alleles with known functional properties are not evident when other alleles with unknown functional properties are also included [12]. Hence only individuals with 4 r/4 r, 4 r/7 r and 7 r/7 r genotypes were included in the analysis.

### Statistical analysis

All statistical analyses were conducted using SPSS 18 (Chicago: SPSS Inc.). Means and standard deviations were computed for all continuous variables. Comparisons between *DRD4*-exIII-VNTR genotype categories were performed using Student's t-tests for continuous variables and Pearson's Chi-square tests for categorical variables. Multiple linear regressions were performed with CD-RISC as continuous outcome variable while controlling for effects of age and sex in all models. Additional covariates such as total years of education and BIS/BAS scales were also included in some models as described below. The continuous variables used in the analyses were not standardized. To test for associations between CD-RISC and the predictor variables of interest, i.e. *DRD4*-exIII-VNTR genotype, number of CA events and BIS/BAS measures, we generated regression models for each of these predictors separately. Since the frequency of the homozygous 7 r (7 r/7 r) genotype was very low in our sample the 4 r/7 r and 7 r/7 r genotypes were pooled (referred to as the 7 r+ group) and compared with the 4 r/4 r genotype (referred to as the 7 r− group) and the *DRD4*-exIII-VNTR genotype group was entered in the model as a binary categorical predictor. As the number of individuals reporting more than 5 adversities were very few, the continuous scale for CA (observed range 0–14) was truncated at 5 and scores ≥5 were grouped together to generate a scale with range 0–5 (0 = no adversity to 5 = 5 or more adversities). Since this study was conducted to test a specific hypothesis that *DRD4*-exIII-VNTR interacts with CA to affect emotional resilience, *DRD4*-exIII-VNTR × CA was the only gene-environment interaction examined. The 7 r− group was the reference genotype. To test for this interaction we generated two regression models with and without the BIS/BAS scales as covariates and the *DRD4*-exIII-VNTR genotype, CA and their interaction term as predictors. Regression models were generated by entering the covariates first, followed by the predictors and then the interaction term. Change in *R^2^* value between each step and the *p* value associated with the *R^2^* change were noted. A similar procedure was followed for testing the effect of *DRD4*-exIII-VNTR genotype and CA interaction on BIS/BAS measures. We report only the final regression model with the interaction term included. For simplicity in interpretation we report results that were significant at the stringent α level of 0.01 instead of the more commonly used level of α = 0.05. However all results remained significant when applying Bonferroni corrections at an α = 0.05 level.

## Results

**Figure 1:**
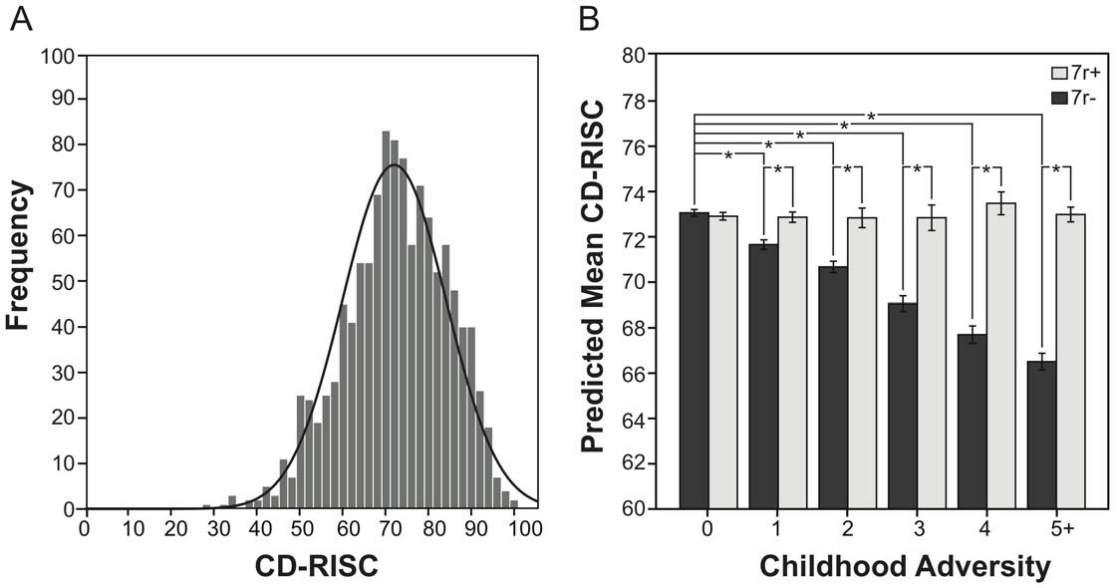
Graphical representation of observed and predicted CD-RISC scores. (A) Distribution of CD-RISC raw scores. (B) Mean values of the CD-RISC scores predicted from the regression equation for different adversity levels. Light and dark bars represent different *DRD4-*exIII-VNTR genotypes as indicated, error bars represent 95% confidence interval and * represent significant result at *p* <0.01.

Sociodemographic characteristics of the sample are reported in Table 1. The measure of resilience, CD-RISC had a mean score of 72.0 with standard deviation of 12.1 in our sample and was approximately normally distributed (Figure 1A) with a left-handed skew since most individuals reported higher than lower levels of resilience (a trend also reported in previous studies [4], [31]). Approximately 45% of participants reported at least one CA and less than 10% reported five or more adversities with domestic conflict reported as the most common form of adversity experienced. Distribution of the genotypes did not differ significantly from the HWE expectation (*p*(likelihood-ratio test)  = 0.907) [12]. Socio-demographic variables, number of CAs and the mean CD-RISC scores were not significantly different between the 7 r+ and 7 r− groups, however a trend was observed with the 7 r+ group reporting a higher mean CD-RISC score compared to the 7 r− group. The difference between the genotype groups with respect to CA was not consistent at all levels of adversity and did not reach statistical significance.

**Table 1:** Demographic characteristics, adversity and resilience measures of individuals with 7 r+ and 7 r− *DRD4*-exIII-VNTR genotypes (mean ± s.d. for continuous variables and frequency for categorical variables shown).

		*DRD4-exIII-VNTR* genotypes		t/χ^2^	df	*p*
		7 r− (n = 676)	7 r+ (n = 472)			
sex				0.060	1	0.807
	Male	303 (44.8%)	215 (45.6%)			
	Female	373 (55.2%)	257 (54.4%)			
age		30.7±1.5	30.7±1.5	−0.108	1146	0.914
education (years)		15.5±1.6	15.3±1.7	1.115	1146	0.265
CA				13.398	5	0.020
	0	285 (42.2%)	229 (48.5%)			
	1	156 (23.1%)	111 (23.5%)			
	2	83 (12.3%)	43 (9.1%)			
	3	54 (8.0%)	29 (6.1%)			
	4	48 (7.1%)	17 (3.6%)			
	≥ 5	50 (7.4%)	43 (9.1%)			
CD-RISC		71.3±12.0	72.9±12.3	−2.281	1215	0.023

t-tests were performed for continuous variables and χ^2^ tests for categorical variables.

CA Childhood adversity.

CD-RISC Connor-Davidson Resilience Scale.

We examined whether in our sample *DRD4*-exIII-VNTR genotype, CA and BIS/BAS scores were significant predictors of CD-RISC (after controlling for age, sex and total years of education) using linear regression (Table 2). While the genotype did not significantly predict CD-RISC scores both CA and personality traits emerged as significant predictors. Greater number of reported CAs was correlated with lower resilience scores. Among the BIS/BAS subscales, BAS-reward response and BAS-drive showed significant positive associations while BIS showed a significant negative association with CD-RISC.

**Table 2:** Multiple regression models with *DRD4-exIII-VNTR* genotype, CA and personality traits predicting CD-RISC.

Predictors		*β*	*p*	R^2^ (change)
*DRD4*-exIII-VNTR^a^		0.070	0.017	0.018 (0.005)
CA		−0.088	0.003^b^	0.021 (0.008^c^)
*personality*				0.172 (0.159^c^)
BAS				
	reward response	0.170	**<0.001** b	
	drive	0.113	**0.001** b	
	fun-seeking	0.070	0.039	
BIS		−0.293	**<0.001** b	

All models controlled for age, sex and years of education. *p* <0.01 shown in bold.

CA: Childhood adversity.

BAS: Behavioral activation system.

BIS: Behavioral inhibition system.

a 7 r− group was the reference genotype.

b significant after Bonferroni corrections at α = 0.05.

c significant R^2^ change from previous model at *p* < 0.01.

To test for gene-environment interactions, we generated different models by regressing CD-RISC on CA, *DRD4*-exIII-VNTR genotype and the interaction term (Table 3). In Model 1 we controlled for age, sex and education and found CA to be a significant predictor with no significant effect of the *DRD4*-exIII-VNTR genotype. We also observed a significant [genotype × CA] interaction. The interaction term appeared to be positively associated with CD-RISC suggesting a protective effect of the 7 r allele in the presence of adversity. These results suggest the following: (i) *DRD4*-exIII-VNTR genotype moderates the effect of CA, (ii) the presence of one or more 7 r allele was not associated with higher resilience when no adversity was reported (there was no difference in mean resilience scores between 7 r− and 7 r+ carriers who reported no adversity) and (iii) the 7 r allele appears to be protective against the decrease in resilience that occurs with increasing adversity (Figure 1B).

**Table 3:** Multiple regression models for interactive effect of *DRD4-exIII-VNTR* genotype and CA on resilience.

	Model 1		Model 2	
Predictors	*β*	*p*	*β*	*p*
*DRD4*-exIII-VNTR^a^	−0.004	0.906	0.028	0.415
CA	−0.158	**<0.001** b	−0.121	**0.001** b
*DRD4* a×CA	0.132	**0.003** b	0.081	0.045
	R^2^ (change): 0.033 (0.008^c^)		R^2^ (change): 0.186 (0.003^c^)	

All models controlled for age, sex and years of education. *p* <0.01 shown in bold.

Model 2 also controlled for BAS/BIS measures.

CA Childhood adversity.

a 7 r− group was the reference genotype.

b significant after Bonferroni corrections at α = 0.05.

c significant R^2^ change from previous model at *p* <0.01.

We then examined whether the moderating effect of the genotype could be detected when other predictors of resilience like BIS/BAS scores were included in the model. Interestingly, when BIS/BAS scores were controlled for in the regression analysis, the effect of the [genotype × CA] interaction term was no longer significant (Model 2, Table 3). These results suggest that the variance in CD-RISC explained by *DRD4*-exIII-VNTR genotype by CA interaction might be explained by BIS/BAS scores. Since previous studies had reported a moderating effect of the *DRD4*-exIII-VNTR genotype on temperament and externalizing behaviour (related to personality traits) in the context of parenting quality (childhood environment [7], [10]) we examined whether BIS/BAS sensitivities were also affected by *DRD4*-exIII-VNTR genotype and CA interaction. We found no main effects of either CA or the genotype on the BAS subscales but a significant interactive effect of the variables (Table 4). There was a significant main effect of CA on BIS but no effect of genotype or the interaction term on this scale (Table 4). Thus *DRD4*-exIII-VNTR genotype moderates the effect of CA on BAS but not BIS sensitivity with individuals carrying one or more 7 r alleles reporting higher behavioral activation compared to those without the 7 r allele but only if they had experienced adversity in childhood. Among individuals who reported experiencing one or more CA, we observed that those carrying the 4 r/4 r *DRD4*-exIII-VNTR genotype had both reduced resilience and lower BAS sensitivity. In contrast, carriers of one or more 7 r alleles have resilience levels and BAS scores comparable to those of individuals who experienced no adversity. Thus the *DRD4*-exIII-VNTR genotype moderates the effect of CA on both resilience and personality traits. Since the BIS/BAS and CD-RISC scores were significantly associated with each other it suggests that the protection in resilience seen in the 4 r/7 r and 7 r/7 r carriers against the effects of CA could be mediated by their greater BAS sensitivities.

**Table 4:** Multiple regression models for interactive effect of *DRD4-exIII-VNTR* genotype and CA on personality traits.

	BAS-reward		BAS-drive		BAS-fun seeking		BIS	
	*β*	*p*	*β*	*p*	*β*	*p*	*β*	*p*
*DRD4*-exIII-VNTR^a^	−0.036	0.333	−0.011	0.767	−0.053	0.158	0.074	0.032
CA	−0.037	0.333	0.029	0.445	0.005	0.897	0.013	**0.001** b
*DRD4* a×CA	0.096	0.029	0.073	0.098	0.115	**0.009** b	−0.070	0.085
	R^2^ (change): 0.026 (0.004)		R^2^ (change): 0.018 (0.002)		R^2^ (change): 0.025 (0.006^c^)		R^2^ (change): 0.175 (0.002)	

All models controlled for age and sex. *p* <0.01 shown in bold.

CA Childhood adversity.

a 7 r− group was the reference genotype.

b significant after Bonferroni corrections at α = 0.05.

c significant R^2^ change from previous model at *p* <0.01.

## Discussion

In this study we investigated gene-environment interaction involving the *DRD4* gene and CA on emotional resilience in young-adults. We have replicated the finding that self-reported experience of adversity during childhood is associated with reduced emotional resilience in adult life [4]. At the group level the CD-RISC score was higher for the 7 r+ compared to 7 r− group but this difference was not statistically significant. The number of CAs reported also appears to differ between these two groups. While this might suggest a possible gene-environment correlation, the difference was not consistent at all levels of adversity and did not reach statistical significance. Furthermore, we have demonstrated that the negative association between CA and emotional resilience is moderated by *DRD4*-exIII-VNTR genotype such that individuals carrying the 7 r allele appear to be protected against a decrease in resilience levels after experiencing adversity in childhood. Previous reports have associated the 7 r allele with differential susceptibility thereby making individuals more responsive to both positive and negative environmental influences [8]. Individuals carrying the 7 r allele were reported to have the best outcomes in a nurturing environment but were also most adversely affected in an unsupportive environment [7], [8], [9], [10]. In contrast, we observed a protective effect of the allele in presence of adversity and no significant effect when childhood adversity was reported to be absent. Thus in relation to emotional resilience the 7 r allele does not appear to be a risk allele, rather it appears to have a protective effect.

Another interesting observation from our study was the effect of *DRD4*-exIII-VNTR genotype and CA on personality traits as measured by self-reported BIS/BAS scales. CA was associated with higher BIS sensitivity but the effect on BAS was dependent on the *DRD4*-exIII-VNTR genotype. Only 7 r carriers reported high BAS sensitivity even after having experienced adversity during childhood. BAS as postulated by Gray [32], [33] is sensitive to signals of reward and escape from punishment and promotes goal-directed behaviour. Reduced BAS sensitivity has been associated with increased risk of depression [34]. Campbell-Sills et al. [35] and Kasch et al. [36] provided evidence for a direct connection between self-reported BAS sensitivity and depression with higher BAS sensitivity being associated with fewer depressive symptoms. Hence increase in BAS sensitivity is likely to be associated with increased resilience. Our results support this hypothesis and also suggest that the protective effect of the 7 r allele on emotional resilience in the face of adversity could be mediated through the development of personality traits that increase sensitivity to rewards.

The main strengths of this paper are that the study was conducted on a large random sample and the inclusion of potential mediating variables such as personality. Our results remained significant after correcting for multiple comparisons. Also, when testing for the genetic effect we included only specific *DRD4*-exIII-VNTR genotypes with known functional differences. This facilitates conceptualisation of the biological mechanisms underlying the genotype effect. However, there are several limitations to this study, some of which are related to the measures used. Measure of CA was derived from retrospective self-reports and hence might not be completely accurate (e.g. social desirability and current emotional state could introduce biases [37], [38]). However, previous studies suggest that although retrospective reports are imperfect, they are not systematically distorted in a way that inflates associations with mental health problems [37], [39]. The Connor-Davidson's CD-RISC scale is a subjective measure of individual perceptions of their ability to recover from adversity and not an objective measure of the true ability. However, previous research has shown that resilience is distinct from both positive and negative affect [22]. In addition, due to the narrow age cohort used in this study, the results need to be replicated in other age groups. It is possible that the genotype effect might not be a result of the VNTR variation but indirect effects of other functional polymorphisms that are in linkage disequilibrium with the VNTR such as the C-521T promoter polymorphism [40].

Despite these limitations our study contributes significantly to the understanding of the effect of CA on resilience in adults by demonstrating the importance of the genetic make-up of individuals, for example their *DRD4*-exIII-VNTR genotype. It brings to light a protective function of the 7 r allelic variant of the well-studied *DRD4*-exIII-VNTR locus, which was not identified in earlier studies. More generally, our results demonstrate how effects of genotypic variation on important health-related phenotypes can depend on its interaction with environmental and life-history variables, with the result that no main effect of the genotype is evident. Genotypic effects of this kind cannot be identified in studies, such as genome-wide association studies, that only examine main effects, or when interactive effects are only investigated once a main effect of the genotype has been identified.
